# Role of HGF–MET Signaling in Primary and Acquired Resistance to Targeted Therapies in Cancer

**DOI:** 10.3390/biomedicines2040345

**Published:** 2014-11-25

**Authors:** Carminia Maria Della Corte, Morena Fasano, Federica Papaccio, Fortunato Ciardiello, Floriana Morgillo

**Affiliations:** Medical Oncology, Department of Experimental and Internal Medicine “F. Magrassi e A. Lanzara”, Second University of Naples, Napoli 80131, Italy; E-Mails: carmydellacorte@gmail.com (C.M.D.C.); morenafasano@ymail.com (M.F.); fede.papaccio@yahoo.it (F.P.); fortunato.ciardiello@unina2.it (F.C.)

**Keywords:** MET, primary and acquired resistance, targeted therapy

## Abstract

The Hepatocyte growth factor (HGF)—mesenchymal-epithelial transition (MET) pathway is deregulated in several cancers and is associated with aggressive phenotype and worse prognosis. MET, a tyrosine kinase receptor activated by HGF, plays a physiological role in embryogenesis, promoting cell growth, survival and motility. HGF–MET aberrant activation in tumorigenesis acts through various mechanisms: paracrine/autocrine HGF production, MET overexpression, MET germ-line and sporadic mutations and cross-talk with other growth factor receptors. In addition, MET activation could represent a mechanism of escape from other targeted therapies, through receptor amplification or over-stimulation by the ligand, as demonstrated in non-small cell lung cancer (NSCLC) and colorectal cancer (CRC) models with acquired resistance to epidermal growth factor receptor (EGFR) inhibitors and also in models of melanoma resistant to the BRAF inhibitor vemurafenib. As a consequence, a lot of molecules targeting MET signaling are under clinical investigation as single agent or in combination with other targeted drugs. Patient selection, based on MET expression on tumor samples (eventually, by re-biopsy of new metastatic sites), and pharmacokinetic/pharmacodynamic markers are needed. Authors review the latest data on the role of MET and the molecular mechanism underlying primary or acquired resistance to biological agents, focusing on NSCLC, CRC and melanoma.

## 1. Introduction

The mesenchymal-epithelial transition factor (*MET*) gene encodes for a membrane-bound receptor tyrosine kinase (RTK) expressed predominantly by epithelial cells. The MET receptor, once activated by its physiological ligand, the hepatocyte growth factor (HGF), stimulates a complex system of intracellular signaling cascades: Src, extracellular signal-regulated kinase 1 and 2 (ERK1/2)/mitogen-activated protein kinases (MAPK), phosphoinositide 3-kinase (PI3K), protein kinase B (AKT), signal transducer/activator of transcription (STAT), nuclear-factor-κB, and mammalian target of rapamycin (mTOR) [1].

A large number of evidences highlights the HGF–MET physiological role in different phases of embryonic development. In particular, in early stages [2], HGF and MET are co-expressed by progenitor cells, indicating an autocrine mechanism of stem cell survival [3]. However, they are also crucial in later moments for embryonic nervous system evolution, epithelial branching morphogenesis and migration of muscle cells progenitors [4] as demonstrated by death *in utero* of HGF or MET null embryos [5]. A key-point is the Epithelial-To-Mesenchymal (EMT) transition induced by HGF–MET interaction, which is critical to guide cells motility and relocation during early development and organogenesis [6].

HGF–MET axis displays also a physiological role in tissue repair: following injury it is essential for protection, regeneration, and anti-fibrotic activity of cutaneous, pulmonary, hepatic, and gastrointestinal tissues [7,8].

As a counterpart, aberrant activation through amplification/overexpression or activating mutations of the HGF–MET signaling cascade is heavily implicated in tumorigenesis of several types of solid tumors [9].

For example, early reports evidenced that germline activating mutations of the tyrosine kinase domain, leading to a constitutive activation of MET receptor, represent a mechanism implicated in the carcinogenesis of hereditary papillary renal cell carcinoma [10], while sporadic MET mutations were involved in glioblastomas, head and neck squamous cell carcinomas and gastric carcinoma [11]. Interestingly, MET expression correlates with esophageal metaplasia–dysplasia–adenocarcinoma transformation [12], with a percentage of MET amplification in about 9% of cases [13]. Aberrant autocrine or paracrine stimulation of the HGF–MET axis contributes to tumorigenesis of breast cancers, osteosarcomas, melanomas [14,15] and gliomas [11].

Taken together, these findings elucidate the MET leading role in tumorigenesis, further confirming its role as proto-oncogene.

Moreover, in preclinical models MET over-expression correlates with worse prognosis and more aggressive phenotype, sustained by cancer cell growth maintenance and progression through hypoxia-induced tumor cell invasion and metastatization [16]. In addition, non-neoplastic cell lines, induced to have constitutive activation of HGF/MET, acquire tumorigenic ability* in vivo* [17,18].

These data are confirmed in several clinical settings: in NSCLC patients, high levels of MET protein correspond to worse clinical outcomes and represent a primary mechanism of resistance to EGFR inhibitors [19]; MET overexpression suggests increased metastatic potential and negatively influences patient survival in hepatocellular carcinoma [20], represents a human epidermal growth factor receptor 2 (HER2) independent negative prognostic marker for node-positive breast cancer [21] and a marker of invasive behavior of CRC [22]. Thus, deregulation of HGF–MET pathway drives progression of various human solid tumors [23].

In recent years, specific oncogenes harboring particular mutations or gene alterations have been discovered as drivers of human cancers and are potentially targetable with new molecular agents: inhibitors of the human epidermal receptors family (HER family) are the most widely developed targeted drugs in NSCLC, breast cancer and CRC treatment. Tumor responses to these drugs are reasonably higher than those obtained with conventional chemotherapy. As a consequence of this molecularly targeted therapeutic approach, the median progression free survival (PFS) of these patients has also significantly improved; however, most if not all of these patients experience disease progression due to innate or acquired resistance. The complex interactions among RTKs are one of the principal mechanisms of resistance to both anti-EGFR/HER1 monoclonal antibodies (cetuximab or panitumumab in CRC) and small inhibitors of the ATP binding pocket of the EGFR receptor (erlotinib and gefitinib in NSCLC). Recent findings evidenced a synergistic interaction between MET and other RTKs, like EGFR, vascular endothelial growth factor receptor (VEGFR) and insulin-like growth factor-1 receptor (IGF-1R), promoting cancer invasiveness and resistance to chemotherapy [24,25]. *In vitro* experiments revealed ligand-independent MET activation through EGFR-MET co-immunoprecipitation in hepatocellular carcinoma cells [26] and promotion of metastatization by MET and IGF-1R cross-talk in pancreatic cancer [27]. Similarly, the role of RTKs and MET has emerged as one of the resistance mechanisms to the BRAF inhibitor (vemurafenib) in melanoma.

Overcoming the resistance to molecularly targeted agents toward personalized cancer treatment strategies is now a new interesting field of research in medical oncology.

## 2. Role of MET in Mediating Resistance to Targeted Therapies in Human Cancers: The Examples of Lung Cancer, Colorectal Cancer and Melanoma

### 2.1. Lung Cancer

Despite the poor long-term outcome of lung cancer patients, a prolonged survival rate has been seen in some subgroups of patients in recent years. The most important reason of this slow improvement is the heterogeneity of NSCLC in terms of both histological and molecular features. Indeed comprehensive genome-wide studies have demonstrated recurrent genetic and epigenetic changes displaying oncogenic properties [28].

The first of these changes has been represented by the discovery of EGFR activating kinase domain mutations, represented by exon 19 in-frame deletion of amino acids 746–750 or exon 21 L858R substitution in EGFR sequence, which identify a subset of patients who can greatly benefit from the treatment with EGFR tyrosine kinase inhibitors (TKIs), gefitinib and erlotinib, which are superior to standard doublet chemotherapy in terms of response rate and PFS [29,30]. However, despite the initially dramatic responses to EGFR TKIs, most of these patients experience disease progression within one year, as a consequence of acquired resistance.

Cellular mechanisms of *de novo* and acquired resistance to EGFR TKIs therapy in NSCLC include various molecular abnormalities in tumor cells: increased angiogenesis, constitutive activation of transducers located downstream to EGFR, overexpression of other tyrosine kinase receptors or secondary EGFR mutation. Among patients exhibiting acquired resistance, a secondary EGFR exon 20 mutation (T790M) occurs in about 50% of cases [31] and an amplification of MET has been detected in up to 20% of EGFR TKI-resistant tumors [32].

In most MET-amplified cancers, MET signals are mediated through human epidermal growth factor receptor 3 (HER3/ErbB3). Indeed, in recent* in vitro* studies, conducted on the human NSCLC HCC827 cell line (harboring the activating *EGFR* mutation in exon 19) and made resistant to gefitinib, revealed that MET became amplified and caused resistance to TKI through an ErbB3-dependent activation of PI3K [33,34]. ErbB3 is the unique member of HER family believed to be “kinase dead”: once heterodimerization occurs with other ErbB family member, ErbB3 is phosphorylated on tyrosines and serves as a scaffold to activate downstream signaling [34]. In lung cancers that are sensitive to EGFR inhibitors, ErbB3 mediates PI3K activation; thus, ErbB3 redundant activation, induced by MET amplification, enables cancer cells to transmit the same downstream signaling even in the presence of EGFR inhibitors and concomitant inhibition of both EGFR and MET is required to kill these resistant cells.

Clinical trials have been conducted in recent years to test anti-MET agents, both TKIs and antibodies, for the treatment of NSCLC patients. In 2011 in a randomized phase II trial was evaluated the activity of the combination of Tivantinib (ARQ197), a highly selective MET TKI, with erlotinib* versus* erlotinib plus placebo in advanced NSCLC patients, at progression on first-line chemotherapy [35]; results showed a trend in favor of the experimental arm in terms of median PFS, which was the primary endpoint of the study (3.8 months for tivantinib* vs.* 2.3 months for the placebo, HR = 0.81, *p* = 0.24), although not statistically significant. Unfortunately, the following phase III MARQUEE study (NCT01244191), comparing the same treatment in non-squamous NSCLC patients was early stopped because of negative results on overall survival (OS), the primary endpoint, at a planned interim analysis [36]. Similarly, the efficacy of onartuzumab (Met–MAb), a humanized monovalent (1-armed) anti-MET antibody, acting through inhibition of HGF binding, was evaluated in a phase II study. The combination of erlotinib and onartuzumab *versus* erlotinib alone was studied in second line therapy of NSCLC patients [37]: among 137 patients randomized, 52% were MET positive at immunohistochemistry (IHC) analysis and experienced a statistically significant reduction in the risk of disease progression by the addition of onartuzumab, (median, 1.5 *vs.* 2.9 months; HR: 0.53; *p* = 0.04), while in MET IHC-negative tumors onartuzumab was detrimental. However, the following phase III study, the MetLung study (NCT01456325) in the same setting of patients did not confirm these positive data.

### 2.2. Colorectal Cancer

CRC is the third leading cause of cancer related deaths worldwide [38,39]; many preclinical and clinical evidences suggested that EGFR aberrant signaling is implicated in progression of CRC, through activation of downstream pathways of ERK1/2 and AKT leading to cell proliferation and survival. Anti-EGFR monoclonal antibodies, cetuximab and panitumumab, are currently used for stage IV CRC treatment in combination with chemotherapy, producing a benefit in terms of OS as compared to chemotherapy alone. These antibodies act binding to the EGF receptor on the cell surface and avoiding its interaction with physiological ligand and dimerization, with consequent inhibition of intracellular signal transduction pathway [40,41].

Cetuximab and panitumumab are effective only in a minority of patients with metastatic CRC, because of primary resistance [42,43,44,45] dependent on V-Ki-ras2 Kirsten rat sarcoma viral oncogene homolog (*KRAS*) gene activating mutations, which prevent both anti-EGFR antibodies from working effectively [46,47,48,49]. Mutations in *KRAS* gene codons 12 and 13 were the first recognized mechanisms of primary resistance to anti-EGFR therapy and all patients are now screened for KRAS mutations prior to administration of anti-EGFR targeted therapies [50]. More recently, mutations affecting other codons of KRAS (61, 117 and 146) as well as mutations in the related oncogene Neuroblastoma RAS viral oncogene homolog (NRAS) have been found [48] and validated as biomarkers of resistance to cetuximab or panitumumab [51].

However, even RAS wild type (RAS without mutations) patients can be anti-EGFR non-responders, due to additional mechanisms of intrinsic resistance, involving all components of EGFR signal transduction pathway, such as mutations in BRAF or phosphatidylinositol-4,5-bisphosphate 3-kinase, catalytic subunit alpha (PIK3CA) [48,52], amplification of HER2 [53,54], MET activation [55], other KRAS mutations [56] and loss of phosphatidylinositol-3,4,5-trisphosphate 3-phosphatase (PTEN) expression [57]. Even for patients who respond to anti-EGFR therapy, progression within the first year of treatment has been described as a result of acquired resistance [42].

In preclinical models, stimulation of MET receptor by HGF confers resistance to anti-EGFR monoclonal antibodies [58] and sensitiveness to anti-EGFR therapies could be restored by inhibition of both MET [47,58] and EGFR receptors.

Of interest, overexpression of Transforming growth factor alfa (TGF-α), a specific EGFR ligand, induced EGFR–MET interaction, with subsequent MET phosphorylation and activation of downstream effectors in EGFR-independent manner causing cetuximab resistance in colorectal cancer cells [59]. Thus, combined inhibition of EGFR and MET receptors could represent a strategy for preventing and/or overcoming cetuximab resistance in patients with CRC.

HGF/MET mediates resistance to anti-EGFR inhibition mostly through ERK and AKT persistent phosphorylation [59]. In addition, similarly to what happened in NSCLC, among seven CRC patients that progressed after initial response to cetuximab, amplification of MET was discovered in the post-treatment tissues from three of these patients [48]. Rare MET-amplified cells have been found also in CRC tissue samples before anti-EGFR treatment, suggesting that EGFR-targeted therapies may act as a selective pressure to expand a preexisting subclonal population of cancer cells harboring MET amplification.

In a randomized phase Ib/II trial was tested AMG 102 (Rilotumumab, Amgen, Thousand Oaks, CA, USA), a humanized monoclonal antibody directed against HGF [60], in combination with panitumumab* versus* panitumumab plus placebo in KRAS wild-type advanced CRC population [61]. Combination treatment showed a better response rate (RR 31% in experimental arm* versus* 21% in control arm) and median duration of response (DoR 5.1 *vs.* 3.7 months, respectively), and a positive trend in median PFS [62]; however, all the results were not statistically significant, possibly due to a small number of patients and statistical bias of the analysis.

Currently, rilotumumab is no longer under investigation in CRC, even if it could be proposed in selected MET positive tumors independently on KRAS status; we are waiting results of a recently completed phase II randomized study of the anti-MET antibody, onartuzumab, in association with standard first-line chemotherapy in CRC patients [63].

Conversely, retrospective subgroup analysis of phase I/II randomized study proposing biweekly schedule of irinotecan and cetuximab plus the MET TKI tivantinib or placebo, in second line therapy of KRAS wild-type advanced CRC patients, failed to demonstrate a statistically significant increase in PFS [64,65].

### 2.3. Melanoma

Melanoma is a very poor prognosis cancer when diagnosed in advanced stage, with a median survival of 13 months. Treatment options are: chemotherapy with dacarbazine, monoclonal antibody anti- cytotoxic T-lymphocytes-associates antigen 4 (CTLA-4) ipililumab and vemurafenib for melanomas carrying BRAF V600E mutation [66,67,68,69].

Among various RTKs studied in melanoma cells to explore pathways responsible for uncontrolled survival and growth, MET has emerged as one of the topmost mediator of proliferation and invasiveness stimulated by the paracrine or autocrine HGF production [70,71].

*In vitro* studies [72] revealed that melanoma cancer cell lines expressing activated MET are sensitive to the MET inhibitor, SU11274, resulting in decreasing levels of MET, AKT and ribosomal protein s6 kinase (S6) phosphorylation and reduced formation of HGF-induced reactive oxygen species. This was the first study showing an efficacy of SU11274 in melanoma.

In melanoma, V600E BRAF mutation results in constitutive activation of MAPK signaling and aberrant proliferation. PLX4032/vemurafenib, a BRAFV600E inhibitor, demonstrated good clinical activity in patients with BRAFV600E melanoma, although drug resistance occurs in almost all treated patients. Resistance to vemurafenib is associated with Mitogen-activated protein kinase kinase (MEK)-induced MAPK activation, independently from BRAF activation. MET amplification (at 7q21) has been identified as a mechanism of innate resistance to vemurafenib in one melanoma cell line, LM38, opening new possibilities for efficacious combinations of MET and BRAF inhibitors [73].

More recently, Wilson* et al* analyzed* in vitro* and* in vivo* models of BRAF mutant cell lines correlating sensitiveness to vemurafenib and HGF expression. They confirmed that increased HGF reduces response to vemurafenib in BRAF mutant cell lines and similarly MET activation (by MET-agonistic antibody) in xenograft models blocked the anti-proliferative effect of vemurafenib. Moreover, the finding of higher plasma levels of circulating HGF in BRAF mutant melanoma patients suggested a trend toward a worse prognosis for these patients [15].

Apart of the cell autonomous mechanisms of drug resistance, some reports indicate an important role displayed by the stroma in the development of resistance. In particular, secretion of HGF by tumor surrounding stroma has been demonstrated, with the resulting activation of MET pathway leading to resistance to vemurafenib. As a result, the combination treatment with vemurafenib and MET inhibitors could represent a new therapeutic approach [14].

Recently, Webster [74,75,76] investigated the role of DNA elements regulating *MET* gene transcription through chromatin looping, during treatment with vemurafenib. Interestingly, they demonstrated that, upon vemurafenib treatment of melanoma cells, the interaction between the DNA enhancer sequences MET + 63 kb and the MET transcription start site (TSS) significantly increased, suggesting an inducible chromatin looping downstream of BRAF inhibition [76] and identifying the transcription factor MITF as possible looping factor for the MET + 63 kb enhancer. To validate this hypothesis, knockdown of MITF decreased the interaction of MET + 63 kb enhancer with MET TSS, thus blocking about 20% of observed gene expression and preventing innate drug resistance to vemurafenib.

## 3. Research Gaps

MET is considered a promising biomarker of worse prognosis in several human cancers. Prospective studies are needed to explore the potential predictive role of MET for benefit from molecularly targeted anti-MET agents. Preclinical studies identifying mechanisms of resistance to anti-EGFR drugs have been translated into several completed or ongoing clinical trials, including combinations of drugs targeting MET or HGF with known anti-EGFR agents.

So far, however, early results from clinical trials have been disappointing. There are multiple potential reasons for these observations. Many clinical trials are undertaken without prospectively evaluating the specific subpopulation of drug-resistant patients: indeed, patients enrolled in most of these studies had not been selected by MET amplification/activation. So far, most studies have assessed the HGF/MET axis only in tumors at baseline, so excluding cases of secondary HGF/MET activation at metastatic progression and treatment resistance.

Future studies should address the need to biopsy the most recent site of metastatic progression, since targeted therapy directed against HGF/MET may be of considerable value in surmounting both primary and acquired resistance in these selected populations.

In addition, the optimal methods to assess MET are still under investigation, resulting in an inefficacious identification of tumors harboring MET activation.

In conclusion, MET could be used as a “mixed” biomarker: negative prognostic value and positive predictive effect. For future analysis, appropriate selection of patient population who can effectively benefit from MET inhibitory treatment and adequate biomarker evaluation should be encouraged and strongly recommended.
